# Transactions of the Association of American Physicians

**Published:** 1892-05

**Authors:** 


TRANSACTIONS OF THE ASSOCIATION OF AMERICAN PHYSICIANS. Sixth session, held at Washington, D. C., September 22, 23, 24, and 25, 1891. Volume VI. Philadelphia: Printed for the Association. 1891.

The sixth volume of the Transactions of this Association of Eminent Medical Practitioners is similar in style and size to the preceding numbers. The sixth session was held in Washington in connection with and as a part of the second Congress of American Physicians and Surgeons. This volume contains twenty-three titles, most of which have been already published in abstract in the medical journals. They are in the main such as are of interest to practitioners of internal medicine, but there are a few titles that can be read with interest by gynecologists and abdominal surgeons.

Among the latter we may mention a discussion on the Remote Results of the Removal of the Ovaries and Tubes, by Wm. T. Lusk, M. D., Wharton Sinkler, M. D., and James J. Putnam, M. D. We are somewhat surprised in reading this discussion by men who are not only able physicians but profound scholars, to find such an expression as the following: "A recent writer, himself a laparatomist, says, etc." (page 87). Now, if there is any excuse at all for the word laparatomy, which is a misnomer in nine times out of ten when applied to abdominal surgery, we insist that the word laparatomist, as applied to abdominal surgeons, is about the worst mongrel in derivation and meaning that could possibly be constructed. We wish the terms "laparatomy" and "laparatomist" were stricken out of the vocabulary of medicine.

Nerve-Stretching in Inveterate Trigeminal Neuralgia, by James Stewart, M. D., will interest both physicians and general surgeons, while the paper on Intestinal Perforation in Typhoid Fever by Reginald H. Fitch, M. D., is another of the author's important clinical contributions to the study of intestinal surgery. The volume is handsomely printed, and edited by the recorder, Dr. I. Minis Hayes, in a most finished and excellent manner. We are always glad to add the volumes of the Transactions of this Association to our library.

